# Pulmonary interstitial cholesterol crystals associated with diffuse lung cysts in adult: a case report and literature review

**DOI:** 10.1186/s13019-016-0397-z

**Published:** 2016-01-19

**Authors:** Min. Zhang, Hong-Tao Tie, Cheng-Long Wang, Qing-Chen Wu

**Affiliations:** Department of Cardiothoracic Surgery, the First Affiliated Hospital of Chongqing Medical University, Chongqing, 400016 China; Department of Pathology, Chongqing Medical University, Chongqing, China

**Keywords:** Cholesterol crystals, Endogenous lipoid pneumonia, Lung cysts

## Abstract

**Background:**

Cholesterol pneumonitis or endogenous lipoid pneumonia (ELP) result from the accumulation of endogenous cholesterol esters in the lungs, leading to a fibroblastic interstitial inflammatory process, and may be complicated by a secondary bacterial or fungal infection. Striking features were cholesterol clefts in the alveolar and interstitial spaces and alveolar wall-thickening with lymphocytic infiltrations, which was called pulmonary interstitial and intra-alveolar cholesterol granulomas (PICG).

**Case Presentation:**

We report a case of pneumothorax with diffuse lung cysts and pulmonary interstitial cholesterol in a 26-year-old woman. Our case is unique because development of PICG or ELP has been observed in children, but rarely in adult. Most cases could be linked to exogenous sources like inhalation of lipid material or gastroesophageal reflex (GER). In our case, no signs of GER could be discovered. Diffuse lung cysts coexisting with pulmonary interstitial cholesterol crystals are never reported. Additionally, no multinucleated giant cells or granuloma are found pathologically, which make the diagnosis of PICG or lipoid pneumonia difficult.

**Conclusions:**

Pulmonary interstitial cholesterol crystals may develop gradually and evenly distributed throughout the entire lung and resulted in severe distortion of the native structure of the lung.

## Background

Cholesterol pneumonitis or endogenous lipoid pneumonia (ELP) was first described by Sullivan in 1961. It results from the accumulation of endogenous cholesterol esters in the lungs, leading to a fibroblastic interstitial inflammatory process, and may be complicated by a secondary bacterial or fungal infection [1]. Histologically, there is an accumulation of lipid-filled macrophages and eosinophilic proteinaceous material derived from degenerating cells, including surfactant from type II pneumocytes, in the alveoli distal to the bronchial obstruction [2]. Other striking features were cholesterol clefts in the alveolar and interstitial spaces and alveolar wall-thickening with lymphocytic infiltrations, which was called pulmonary interstitial and intra-alveolar cholesterol granulomas (PICG). We report a case of pneumothorax with diffuse lung cysts and pulmonary interstitial cholesterol in a 26-year-old woman. Development of PICG or ELP has been observed in children, but rarely in adult. Most cases could be linked to exogenous sources like inhalation of lipid material or gastroesophageal reflex (GER). In our case, no signs of GER could be discovered. Other typical causes like obstruction by a tumor or foreign body were excluded. Coexisting with lung cysts and pneumothorax is not reported yet.

## Case report

**Fig. 1:**
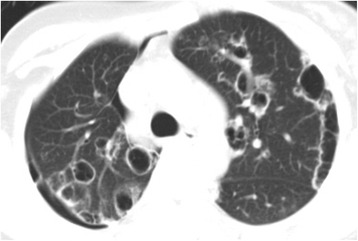
HRCT shows extensive cystic air spaces throughout both lungs with right pneumothorax

**Fig. 2:**
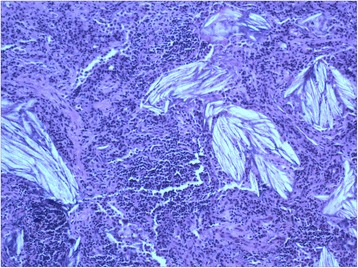
Histopathological findings of the lung biopsy demonstrated numerous lymphocyte infiltration and pulmonary interstitial cholesterol crystals (hematoxylin-eosin staining)

This 26 year old non-smoker woman presented with a spontaneous pneumothorax at 3 months ago. The pneumothorax was treated by simple aspiration resulting in adequate clinical and radiological improvement. However, 3 months later, she presented with further pneumothorax. She had no signs of tuberculosis, rheumatic disease and other background diseases. A lung HRCT scan showed extensive cystic air spaces throughout both lungs on suspicion of lymphangioleiomyomatosis (LAM) (Fig. 1). In view of her age a thoracoscopic lung biopsy was performed in July 2013. At operation there were numerous cystic areas within all lobes of the right lung and she had a large apical bulla. There were no post-operative complications and the lung remained fully expanded. Histology of the lung showed smooth muscle, staining for desmin, HMB-45, β-catenin, SMA, ER, and PR, within the lung, alveolar walls and blood vessels not in keeping with LAM. However, there are diffuse pulmonary interstitial cholesterol clefts surrounded by lymphocytes, without the formation of granulomas (Fig. 2).

## Discussion

Lipoid pneumonia is classified as exogenous or endogenous according to the source of lipid that accumulates in the lungs [3]. Although the most common sources of lipid within the alveoli, bronchioles and interstitial tissues are aspirated and/or inhaled exogenous mineral oils, our patient had no evident history of exposure to mineral oils or other fat-like materials. Besides her clinical history, the diffuse distribution of the lesions in this case appeared to differ from that seen in exogenous lipoid pneumonia, which is usually predominant in the lower and right middle lobes. Therefore, exogenous lipoid pneumonia was considered unlikely in this case. Endogenous lipoid pneumonia, also called “cholesterol pneumonia” or “golden pneumonia”, is an obstructive pneumonitis. It usually develops when lipids that normally are found in the lung tissue escape from destroyed alveolar cell wall distal to an obstructing, usually malignant, airway lesion or from lung tissue damaged by a suppurative process. Cytological examination showed predominantly foam cell macrophages containing large fatty vesicles, and lipid droplets were detected on Sudan III staining, characteristic for lipoid pneumonia. This is not identified in our case. Less commonly, endogenous lipids appear in the lung with fat embolism or thromboembolism, Wegener’s granulomatosis, pulmonary alveolar proteinosis, or lipid storage diseases. Berghaus and colleagues [4] reported a case of endogenous lipoid pneumonia associated with primary sclerosing cholangitis, reveals that hypercholesterolaemia can be the underlying cause of an ongoing inflammatory process. Our patient had a normal serum cholesterol concentration, however. This indicates that the activity of the disease is not necessarily mirrored by the serum total cholesterol concentration, which might explain the discrepancy of the severity of disease in our patient and the normal serum cholesterol concentration.

## Conclusions

The present case was unique because diffuse lung cysts coexisting with pulmonary interstitial cholesterol crystals are never reported. Additionally, no multinucleated giant cells or granuloma are found pathologically, which make the diagnosis of PICG or lipoid pneumonia difficult. The underlying relationship between lung cysts and pulmonary interstitial cholesterol crystals are unclear. We speculated that both are idiopathic manifestations of the same disease. Pulmonary interstitial cholesterol crystals may develop gradually and evenly distributed throughout the entire lung and resulted in severe distortion of the native structure of the lung. This distortion may explain the cause of lung cysts.
